# Initiation of tumor dormancy by the lymphovascular embolus

**DOI:** 10.18632/oncotarget.28658

**Published:** 2024-10-11

**Authors:** Yin Ye, Justin Wang, Michael G. Izban, Billy R. Ballard, Sanford H. Barsky

**Affiliations:** ^1^Department of Pathology, Anatomy and Cell Biology and the Clinical and Translational Research Center of Excellence, Meharry Medical College, Nashville, TN 37208, USA; ^2^Department of Graduate Medical Education, Scripps Mercy Hospital, San Diego, CA 92103, USA

**Keywords:** dormancy, lymphovascular embolus, mTOR, E-cadherin proteolysis

## Abstract

Cancer dormancy followed by recurrence remains an enigma in cancer biology. Since both local and systemic recurrences are thought to emanate from dormant micrometastasis which take origin from lymphovascular tumor emboli we wondered whether the process of dormancy might initiate within lymphovascular emboli. This study combines experimental studies with a patient-derived xenograft (PDX) of inflammatory breast cancer (Mary-X) that spontaneously forms spheroids *in vitro* and budding lymphovascular tumor emboli *in vivo* with observational studies utilizing tissue microarrays (TMAs) of human breast cancers. In the experimental studies, Mary-X during both lymphovascular emboli formation *in vivo* and spheroidgenesis *in vitro* exhibited decreased proliferation, a G_0_/G_1_ cell cycle arrest and decreased mTOR signaling. This induction of dormancy required calpain-mediated E-cadherin proteolysis and was mediated by decreased P13K signaling, resulting in decreased mTOR activity. In observational human breast cancer studies, increased E-cadherin immunoreactivity due to increased E-cad/NTF-1 but both decreased Ki-67 and mTOR activity was observed selectively and differentially within the lymphovascular tumor emboli. Both our experimental as well as observational studies indicate that *in vivo* lymphovascular tumor emboli and their *in vitro* spheroid equivalent initiate dormancy through these pathways.

## INTRODUCTION

Cancer dormancy, followed by recurrence remains a poorly understood phenomenon in both cancer biology and oncology [1–7]. In patients, dormancy refers to the period between initial cancer detection, treatment and remission and its recurrence months to years later [8–12]. This dormancy interval is also termed latency and differs for different cancers and different populations [13]. For example most cases of colorectal cancer recur earlier than cases of breast cancer, matched stage for stage [14–16]. Although some cancers during their latency period are still treated with adjuvant therapy (hormonal, chemo or immunotherapy), the vast majority of cancers during dormancy undergo only active surveillance or expectant management [17].

Relapse from cancer dormancy can occur either locally (near the site of the primary cancer) or systemically (metastatic site) [18]. The epicenter for both is thought to be the so-called micrometastasis, a clump of tumor cells that has escaped the confines of the primary cancer through the phenomenon of lymphovascular invasion, a step which is also poorly understood and a step which some have called, “a metastasis caught in the act” [19]. We therefore wondered whether a greater understanding of the lymphovascular tumor embolus and its signaling pathways might shed light not only on cancer relapse or the release from dormancy but also on the initiation of dormancy in the first place.

Previous studies by us and others using patient-derived xenografts (PDX) of inflammatory breast cancer, including Mary-X had shed some light on the mechanisms responsible for the genesis of the lymphovascular tumor embolus [20–28].

In addition, we had collected a number of cases of human breast cancer of all types including inflammatory breast cancer (IBC) and non-IBC that exhibited lymphovascular emboli that could be subjected to tissue microarray (TMA) algorithmic image analysis [29–33]. Using both experimental and observational studies that focused on the lymphovascular tumor embolus per se, we investigated whether the lymphovascular tumor embolus initiated dormancy and the possible mechanisms behind such initiation.

## RESULTS

### Experimental studies

#### Growth studies on Mary-X and Mary-X Spheroids

**Figure 1:**
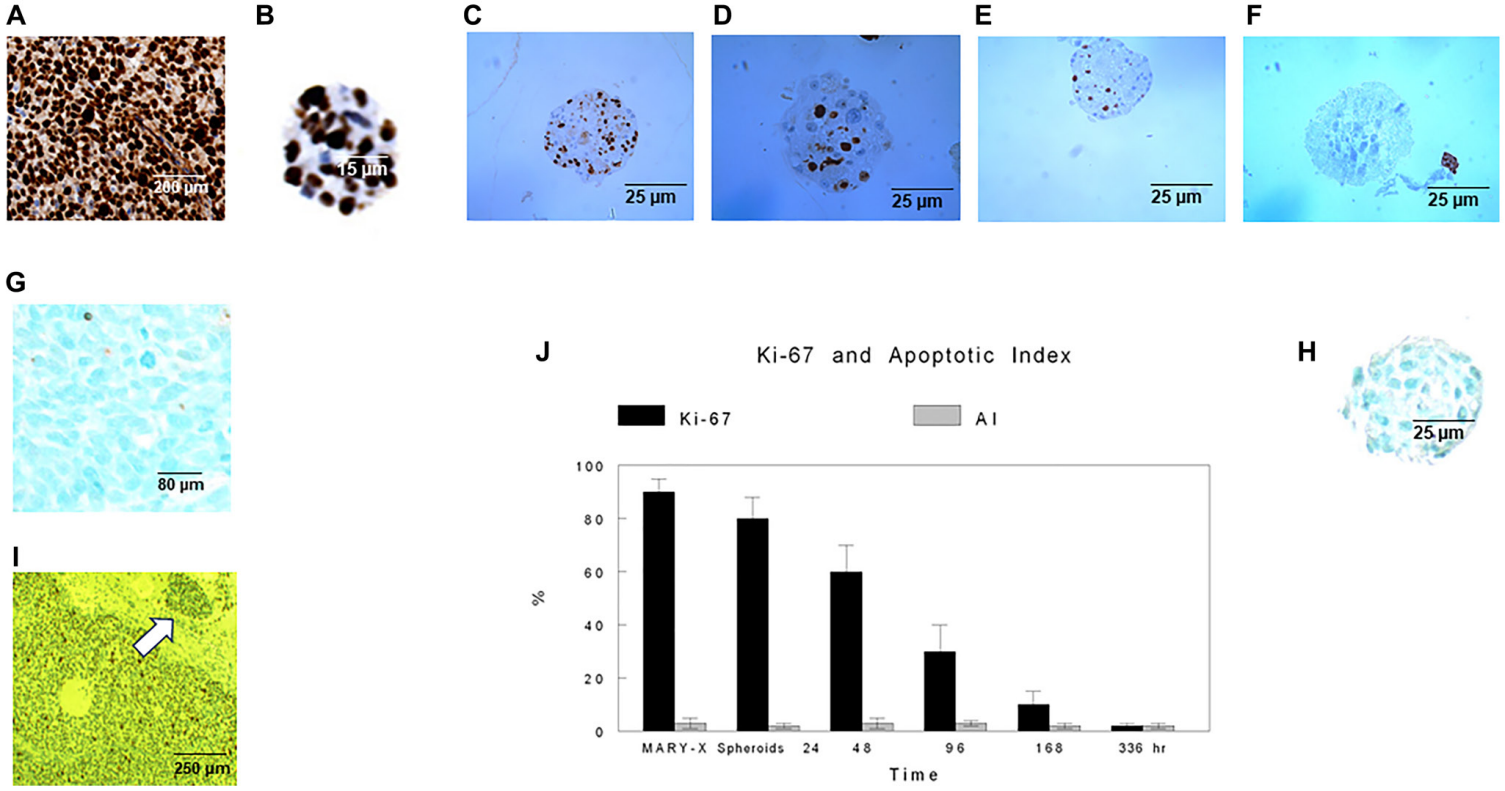
PI and AI IHC. Ki-67 (PI) and AI (TUNEL) IHC depicted in Mary-X and Mary-X spheroids in suspension culture for various time periods (**A**–**H**). Ki-67 decreased within the lymphovascular emboli of Mary-X (see arrow) (**I**) as well as in the spheroids over time (B–F, **J**) whereas AI remained low and unchanged (H, J). Scale bars are provided. For each of these parameters, the graph (J) depicts calculated mean ± SD of 100 spheroids at each time point. Differences of significance are depicted.

Mary-X exhibited a very high proliferative index (PI) of Ki-67 immunoreactivity (Figure 1A). Its lymphovascular tumor emboli, however, showed a decreased PI (*p* < .05) (Figure 1I). The apoptotic index (AI), however, was quite low within both its lymphovascular tumor emboli as well as its non-embolic areas (2–3%) (*p* > .1) (Figure 1G). Mary-X spheroids, which by Principal Component Analysis, had been shown to be the *in vitro* equivalent of lymphovascular tumor emboli [34, 35] continued to exhibit a high proliferative index which then decreased fairly rapidly, reaching an index of nearly 0 after 2 weeks in suspension culture (Figure 1B–1F, 1J). Their AI remained low at 2% (Figure 1H, 1J). Though the spheroids showed a PI of 0 after 2 weeks, they also showed no increase in non-apoptotic cell death assessed by SYTOX Green staining (data not shown). The spheroids remained viable over the six month period of study and were able to again grow into Mary-X when reinjected into mice.

### Cell cycle (flow cytometric) studies of Mary-X spheroids

**Figure 2:**
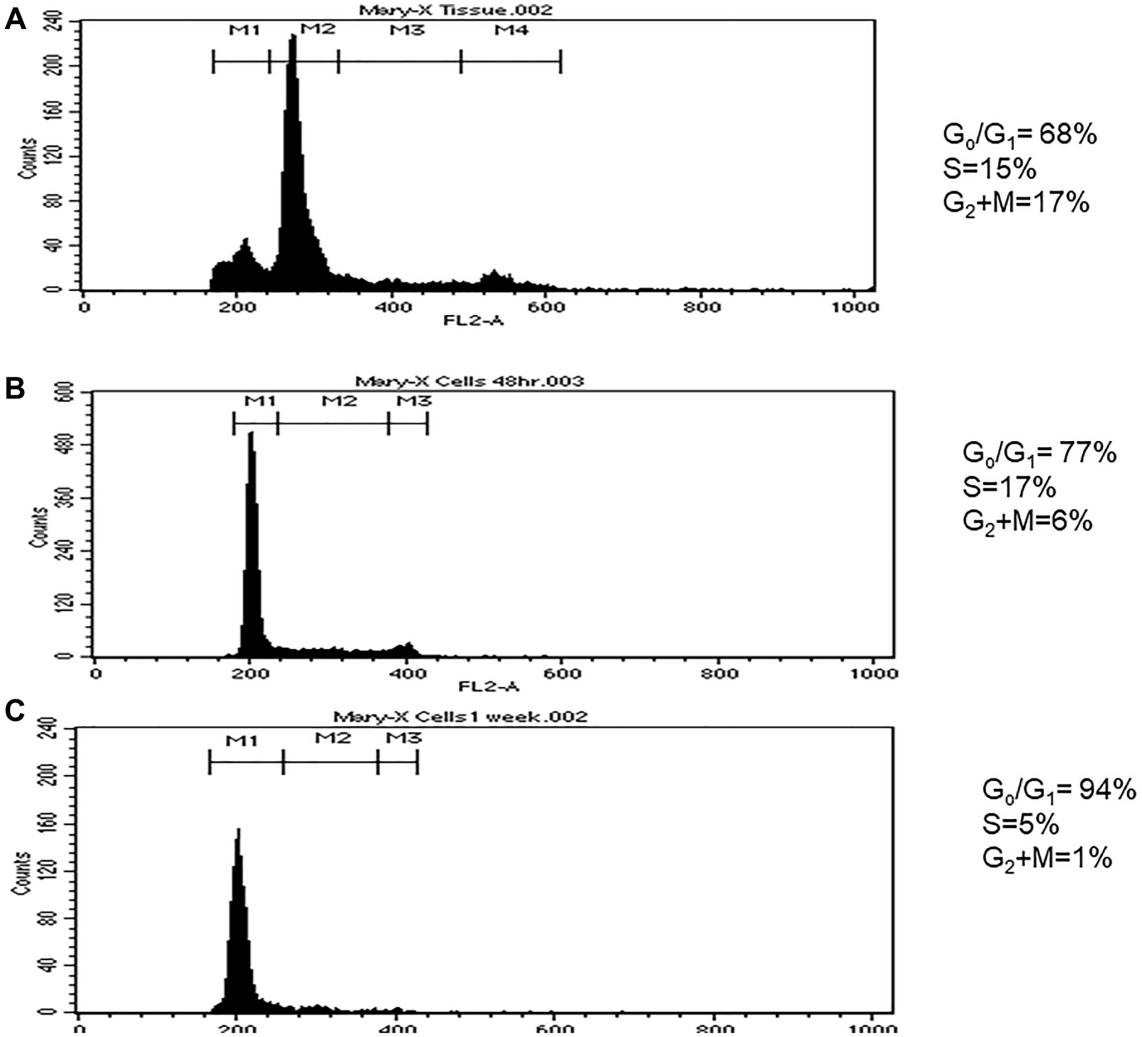
Cell cycle histograms. Cell cycle (DNA ploidy) histogram of Mary-X (**A**), Mary-X spheroids at 48 hr (**B**) and Mary-X spheroids at 168 hr (1 week) (**C**). A progressive G_0_/G_1_ arrest was observed.

Cell cycle DNA histograms revealed a G_0_/G_1_ cell cycle arrest which became more prominent in the Mary-X spheroids over time in suspension culture (Figure 2A–2C).

### Metabolic pathway studies of Mary-X and other breast cancer cell lines

#### Progressive decrease of mTOR activity with spheroidgenesis

**Figure 3:**
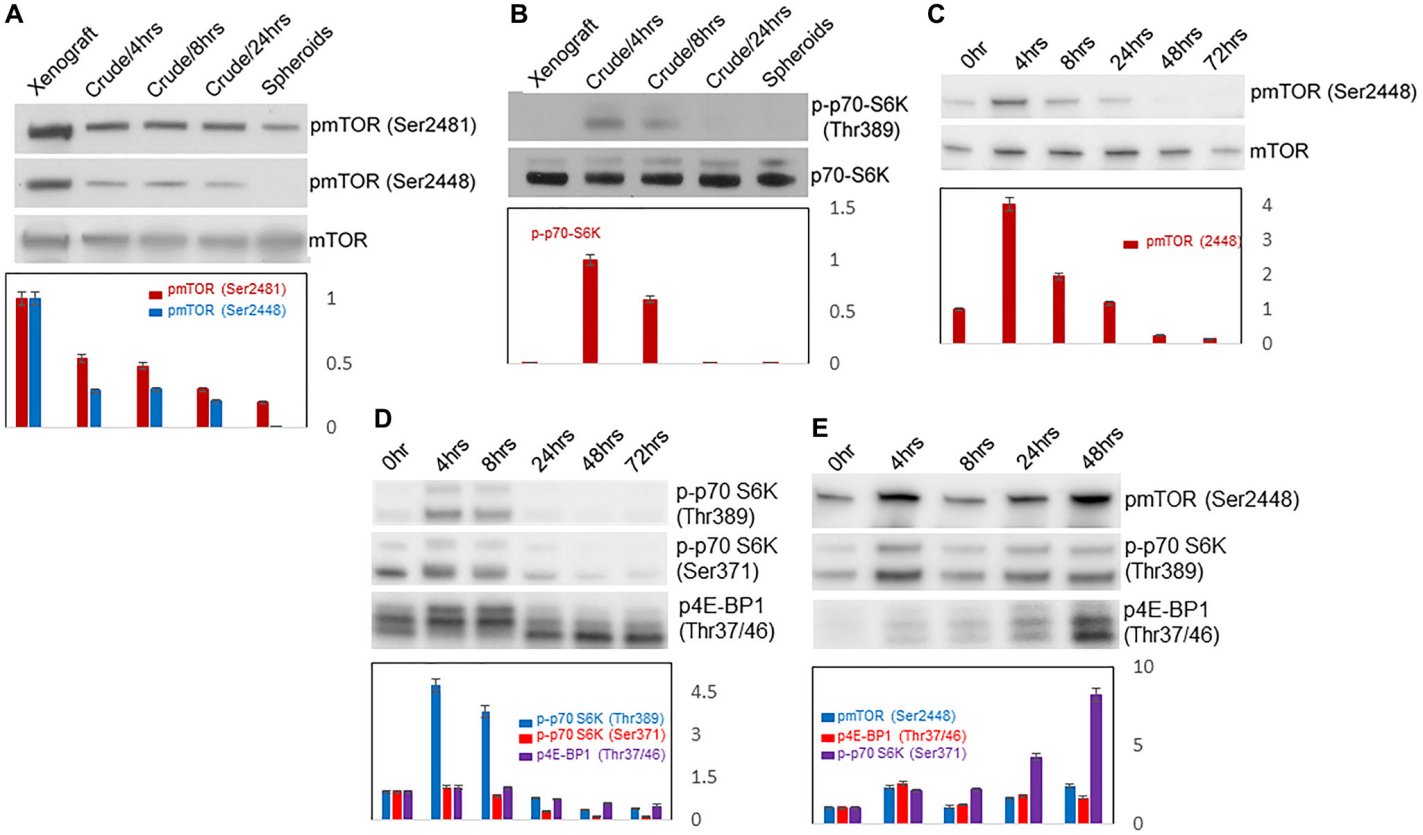
Western blots of mTOR activity. Westen blots of mTOR activity with spontaneous spheroidgenesis of Mary-X as measured by pmTOR (Ser2481) and pmTOR (Ser2448) (**A**); of downstream mTOR substrate p-p70-S6K(Thr389) activity in Mary-X spheroids (**B**); of mTOR activity as measured by pmTOR(Ser2448) in induced MCF-7 spheroids (**C**); of downstream mTOR substrates p-p70-S6K(Thr389), p-p70-S6K(Ser371) and p4E-BP1 in induced MCF-7 spheroids; (**D**); of pmTOR(Ser2448) and downstream mTOR substrates p-p70-S6K(Thr389) and p4E-BP1in induced MCF-7 spheroids (**E**).

We wondered whether this decreased PI and growth arrest during spheroidgenesis while still retaining viability might be accompanied by reduced metabolism which would suggest dormancy, so we first investigated mTOR activity. Our results showed, in fact, that there was a decrease in mTOR activity. The levels of both phosphorylated mTOR (Ser2448) and phosphorylated mTOR (Ser2481) as well as phosphorylated p70-S6K (Thr389), one of the mTOR substrates, also decreased during spheroidgenesis (Figure 3A, 3B). This suggested that their metabolism indeed was reduced.

#### Progressive decrease of mTOR activity with E-cadherin spheroidgenesis

Because our previous studies had demonstrated that calpain-mediated proteolytic processing of E-cadherin specifically into E-cad/NFT1 regulated Mary-X spheroidgenesis [34, 35], we wanted to ultimately see whether this E-cadherin proteolysis also regulated mTOR. More immediately we wanted to see whether the decrease in mTOR activity could also be observed in other E-cadherin-positive cell lines. Because Mary-X was the only model that spontaneously formed spheroids [20, 21], we used other E-cadherin positive cell lines, eg. MCF-7, which not only exhibited calpain-mediated E-cadherin proteolysis but which also could be induced to form spheroids by growing them on ULA plates. Our results confirmed the similar decrease in mTOR activity during induced spheroidgenesis. Initially when MCF-7 cells were digested by trypsin into single cells, the level of phosphorylated mTOR (Ser2448) was low (Figure 3C). Its level then increased and peaked 4 hours after seeding. At that stage, MCF-7 cells proliferated and formed loose aggregates. After that time, the level of phosphorylated mTOR (Ser2448) decreased and reached its lowest levels at 24–72 hr when the induced spheroids reached their highest densities (Figure 3C). Similarly, at the very early stage of induced spheroidgenesis, the levels of phosphorylated p70-S6K (Ser371) and p70-S6K (Ser389) also increased and similarly decreased in later stages of induced spheroidgenesis (Figure 3D); the level of phosphorylated 4E-PB1 (Thr37/46) also decreased in later stages of induced spheroidgenesis (Figure 3D). These collective results demonstrated that in both spontaneous as well as induced spheroidgenesis. mTOR activity decreased. Similar results were also observed in another E-cadherin positive cell line, T47D (data not shown).

However when we investigated these findings in E-cadherin negative breast cancer cell lines, eg., MDA-MB-468, the levels of phosphorylated mTOR (Ser2448), phosphorylated p70-S6K and phosphorylated 4E-BP1 (Thr37/46) all increased throughout induced spheroidgenesis (Figure 3E). Similar results were also observed in another E-cadherin negative cell line, MDA-MB-231 (data not shown).

These results suggested that the presence of E-cadherin was necessary for the decreased mTOR activity which was observed.

#### Progressive decrease of AMPK activity with spheroidgenesis

**Figure 4:**
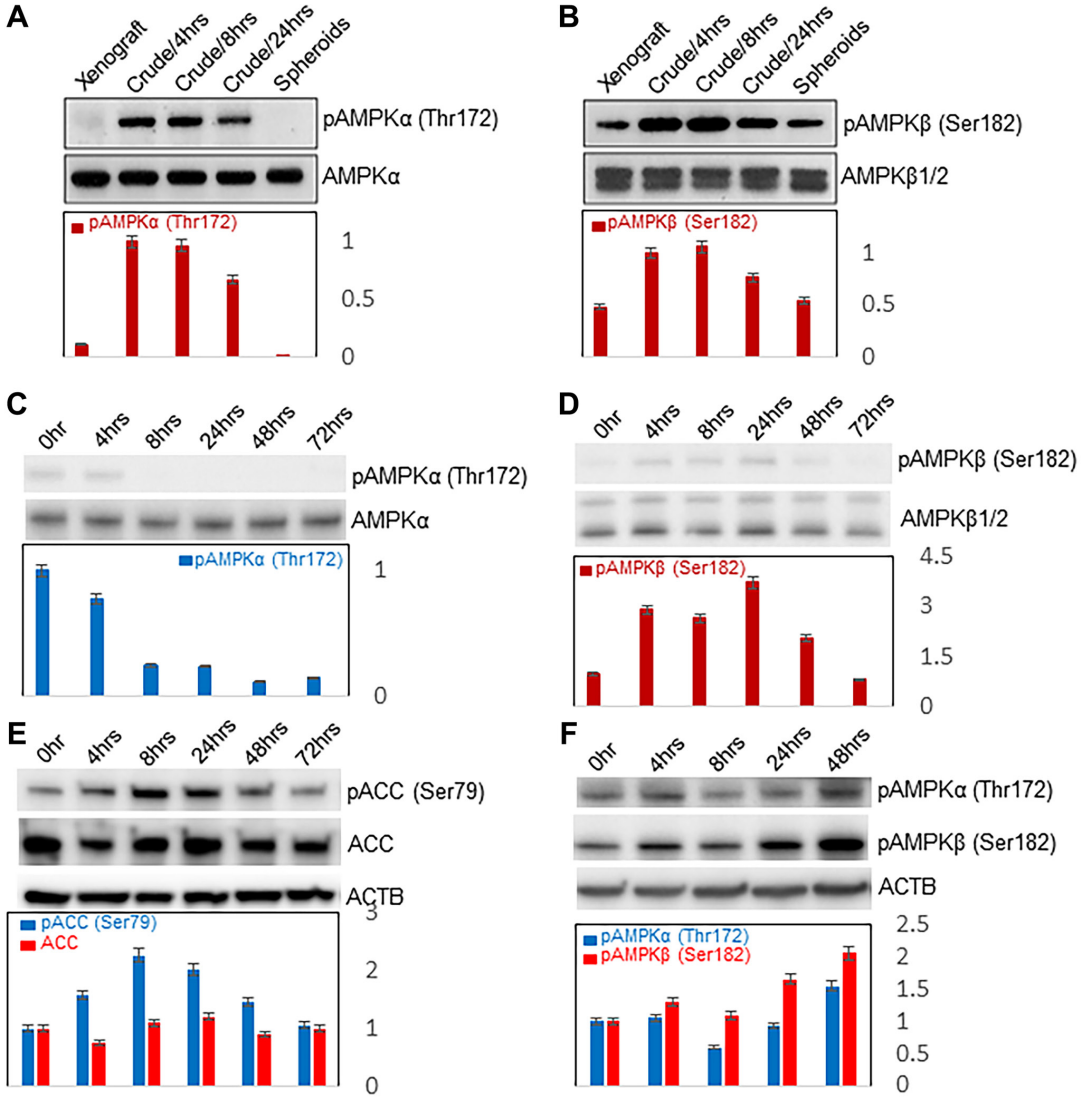
Western blots of AMPK activity. Westen blots of AMPK activity with spontaneous spheroidgenesis of Mary-X as measured by pAMPKα(Thr172) (**A**) and pAMPKβ(Ser182) (**B**); of AMPK activity with induced spheroidgenesis of E-cadherin positive MCF-7 cells as measured by pAMPKα(Thr172) (**C**) and pAMPKβ(Ser182) (**D**); of the downstream ACC pathway as measured by p-ACC(Ser79) (**E**); of AMPK activity with induced spheroidgenesis of E-cadherin negative MDA-MB-468 cells as measured by pAMPKα(Thr172) and pAMPKβ(Ser182) (**F**). ACTB housekeeping probe was used to normalize for protein loading on divided blots depicted in Figure 3E as well as Figure 4F.

AMPK activity also decreased during spontaneous spheroidgenesis of Mary-X (Figure 4A, 4B). While both AMPKα and AMPKβ subunits were stably expressed during Mary-X spheroidgenesis, the levels of activated or phosphorylated AMPKα (Thr172) and AMPKβ (Ser182) subunits decreased at late or end-stage spheroidgenesis when the cellular density of the spheroids was highest (Figure 4A, 4B). We then wanted to see whether the decrease in AMPK activity could also be observed in other E-cadherin-positive cell lines. So AMPK activity was investigated in induced MCF-7 spheroidgenesis. While total AMPKα was stably expressed throughout induced spheroidgenesis, phosphorylated AMPKα (Thr172) decreased during late spheroidgenesis (Figure 4C). Similarly while total AMPKβ was stably expressed throughout induced spheroidgenesis, active or phosphorylated AMPKβ (Ser182) also decreased during late spheroidgenesis (Figure 4D). We next investigated the alterations of the Acetyl-CoA carboxylase (ACC) pathway, a downstream AMPK pathway since active AMPK is thought to phosphorylate ACC at Ser79. Phosphorylated ACC (Ser79) decreased in late spheroidgenesis, mirroring the activity of AMPK (Figure 4E). Similar results were observed with the T47D line (data not shown).

We then used the E-cadherin-negative breast cancer cell line, MDA-MB-468 to investigate the activity of the AMPK pathway in induced spheroidgenesis. The levels of both phosphorylated AMPKα (Thr172) and phosphorylated AMPKβ (Ser182) continued to increase throughout all stages of spheroidgenesis reaching maximal levels at late spheroidgenesis (Figure 4F). Similar results were observed in another E-cadherin-negative breast cancer cell line MDA-MB-231 (data not shown).

These results suggested that the presence of E-cadherin was necessary for the decreased AMPK activity which was observed.

#### Inverse correlation of mTOR and AMPK activities with calpain 2 and its proteolytic E-cadherin fragment, E-cad/NTF1

**Figure 5:**
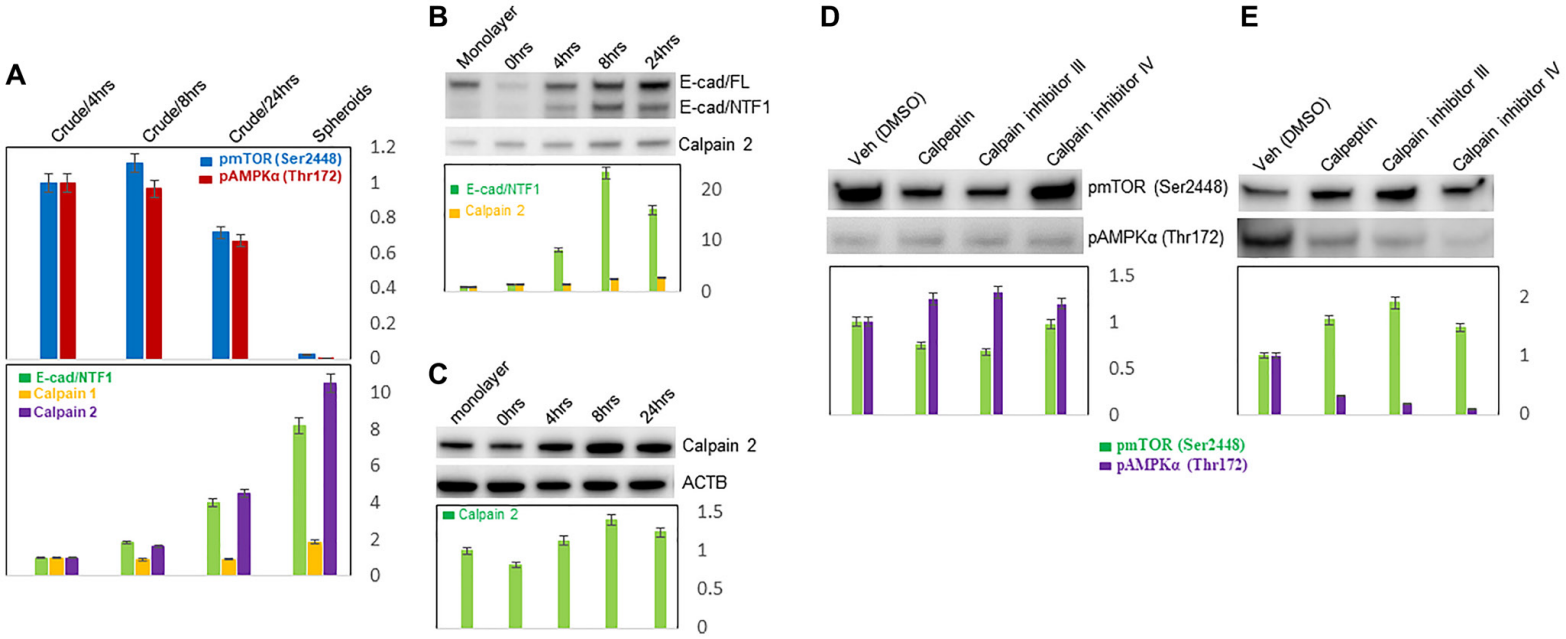
Inverse correlation of E-cadherin proteolysis with mTOR and AMPK activities. Westen blots of mTOR and AMPK activities and their correlation with levels of calpain 2 and E-cad/NTF1 in spontaneous spheroidgenesis of Mary-X (**A**); of calpain 2 and E-cad/NTF1 and their correlation with the stages of induced spheroidgenesis of E-cadherin positive MCF-7 cells (**B**); of calpain 2 and its correlation with the stages of induced spheroidgenesis of E-cadherin negative MDA-MB-468 cells (**C**). Westen blots of the effects of calpain inhibition on mTOR and AMPK activities in MCF-7 cells grown as monolayers (**D**) and as induced spheroids (**E**).

Since our previous studies with Mary-X had shown that a multi-enzyme synergistic cascade of E-cadherin proteolysis involving calpain, especially calpain 2 with the generation of E-cad/NTF1 was required for the formation of both spontaneous spheroidgenesis and lymphovascular emboli formation [34, 35], we wondered whether calpain 2 and its proteolysis of E-cadherin might be responsible also for the decreased mTOR and AMPK activites. The activities of both AMPK and mTOR indeed were both inversely correlated with the levels of calpain 2 and its product E-cad/NTF1 (Figure 5A). In early Mary-X spheroidgenesis, both phosphorylated mTOR (Ser2448, 2441) and AMPKα (Thr172)/β1 (Ser182) remained at high levels. In late or end stage-stage spheroidgenesis, the levels of both phosphorylated mTOR and AMPKα decreased and calpain 2 and its product, E-cad/NTF1, increased.

We again used the E-cadherin-positive MCF-7 to investigate whether the activities of both mTOR and AMPK inversely correlate with the levels of calpain 2 and its proteolytic E-cadherin fragment, E-cad/NTF1 during induced spheroidgenesis. During induced spheroidgenesis, both calpain 2 and calpain-mediated E-cadherin proteolysis (E-cad/NTF1) increased (Figure 5B). In the experiments we had described earlier in this study, both mTOR (Figure 3C, 3D) and AMPK activities (Figure 4C, 4D) decreased over this same time period. We also analyzed the expression of calpain 2 in the E-cadherin-negative line MDA-MB-468 line and found that calpain 2 also increased during induced spheroidgenesis though no E-cad/NTF1 could be generated since E-cadherin was absent (Figure 5C).

#### Alterations in mTOR and AMPK activities with calpain inhibition

Based on the finding that the activities of both mTOR and AMPK were inversely correlated with the levels of calpain 2 and its proteolytic E-cadherin fragment, E-cad/NTF1, during Mary-X spheroidgenesis, we asked whether calpain inhibitors increased both mTOR and AMPK activities in Mary-X spheroids and in fact, they did (data not shown). We used three calpain inhibitors: calpeptin, a cell-permeable calpain inhibitor, which inactivated calpain 1, calpain 2, and papain; calpain inhibitor III, a cell-permeable inhibitor of calpain 1 and 2; and calpain inhibitor IV, a potent, cell-permeable and irreversible inhibitor of calpain 2. We extended our initial studies in Mary-X spheroids to MCF-7 cells growing both as a monolayer and as induced spheroids. In monolayers, calpeptin and calpain inhibitor III led to decreases in the levels of phosphorylated mTOR (Ser2448) but no significant alterations in the levels of phosphorylated AMPKα (Thr172); calpain inhibitor IV resulted in no significant changes in the levels of either mTOR (Ser2448) or AMPKα (Thr172) activities (Figure 5D). However, in induced MCF-7 spheroids, all three inhibitors: calpeptin, calpain inhibitor III and IV led to significant increases in mTOR (Ser2448) activity but surprisingly decreases in the levels of AMPKα (Thr172) activity (Figure 5E). Although we can not explain the opposite effects on increased mTOR (Ser2448) but decreased AMPKα activity in induced spheroidgenesis compared to spontaneous spheroidgenesis where both were increased, it is noteworthy that the regulation of both mTOR and AMPK activities by calpain inhibitors was dramatically altered in the induced spheroids *v* the monolayers, implying that the three-dimensional structure of the induced spheroids influenced the calpain-regulated activities of the two pathways.

#### Phosphoinositide 3-kinase (PI3K) regulation of mTOR pathway

**Figure 6:**
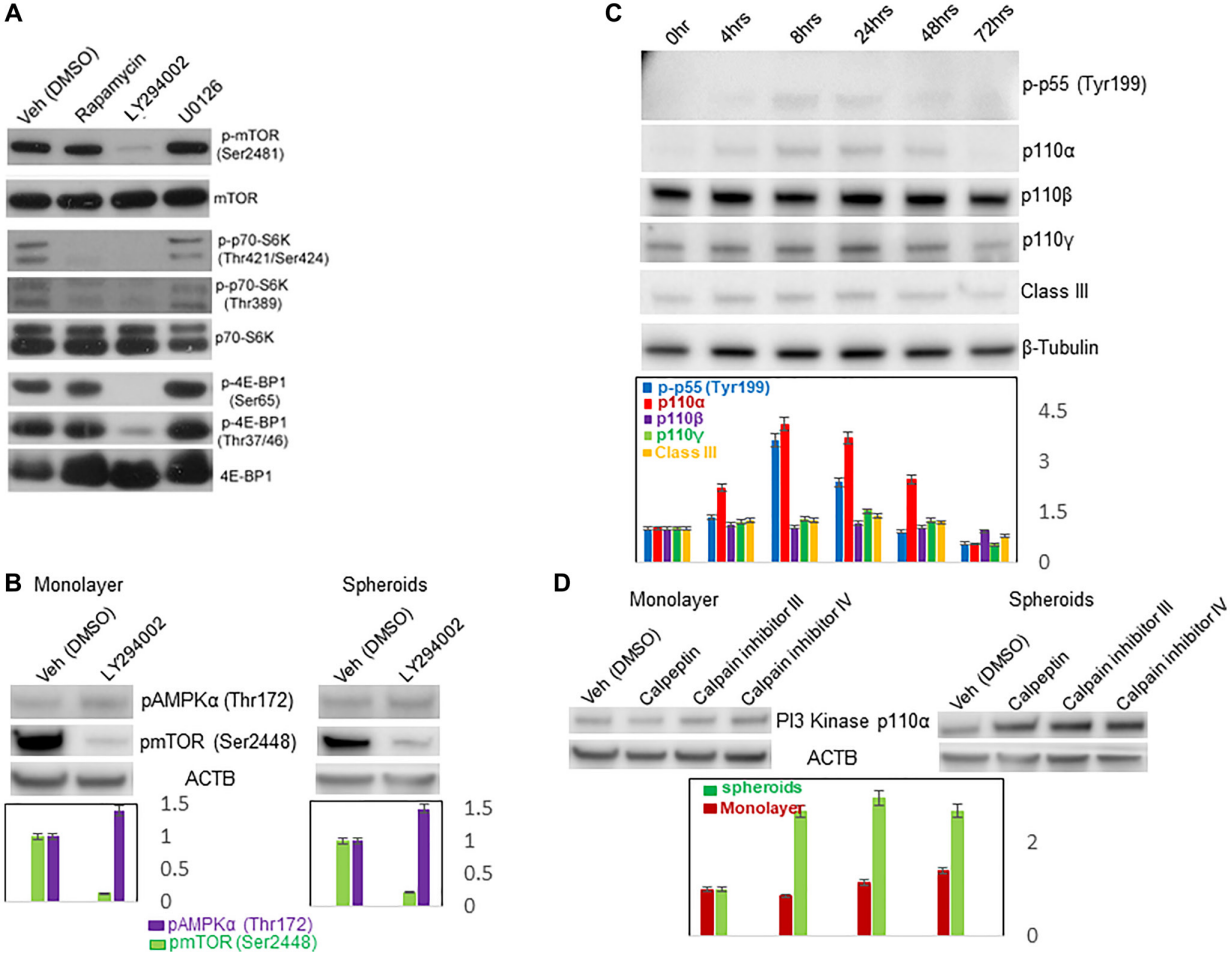
Effects of specific pathway inhibitors on mTOR, AMPK and P13K activities. Westen blots of the effects of different pathway inhibitors including rapamycin (mTOR), LY294002 (P13K) and U0126 (MAPK) on mTOR activity in Mary-X spontaneous spheroidgenesis (**A**); of the opposite effects of LY294002 (P13K) on mTOR and AMPK activities in both MCF-7 monolayers (left) and induced spheroids (right) (**B**); of P13K activity during induced spheroidgenesis of MCF-7 cells (**C**); of the differential effects of calpain inhibition on P13K activity in MCF-7 monolayers (left) *v* induced spheroids (right) (**D**). β-Tubulin housekeeping probe was used to normalize for protein loading on divided blots depicted in Figure 3C, 3D as well as Figure 6C. ACTB housekeeping probe was used to normalize for protein loading on divided blots depicted in Figure 5D, 5E as well as Figure 6D, left and right.

Because the P13K pathway had also been previously implicated in both the regulation of cancer growth as well as metabolism, we investigated this pathway in Mary-X spheroidgenesis. We applied known inhibitors to a variety of different pathways including rapamycin (mTOR), U0126 (MAPK) and LY294002 (P13K) to Mary-X spheroids and examined them at late stage spheroidgenesis. The inhibition of the P13K pathway with LY294002 proved more potent than even rapamycin in the inhibition of mTOR and its downstream substrates (Figure 6A). With P13K pathway inhibition, the levels of both phosphorylated mTOR (Ser 2481) and the substrates of mTOR, phosphorylated p70-S6K (Thr421/Ser424, Thr389) and 4E-BP1 (Ser65, Thr37/46) were dramatically decreased (Figure 6A).

We extended these observations to the induced spheroidgenesis of E-cadherin positive MCF-7 cells. In both MCF-7 monolayers and induced spheroids, inhibition of the P13K pathway by LY294002 similarly led to decreased phosphorylated mTOR (Ser2448). Interestingly this P13K pathway inhibition resulted in increased AMPK activation with increased levels of phosphorylated AMPKα (Thr172) (Figure 6B).

We then examined the activity of the P13K pathway directly in the induced MCF-7 spheroids. Our results showed that induced spheroidgenesis led to decreased PI3K activity with marked alterations in the levels of the phosphorylated regulatory subunits of PI3K. At the start of induced spheroidgenesis when MCF-7 cells were digested by trypsin into single cells, the levels of both p110α and p-p55(Tyr199) were low (Figure 6C). Their levels increased and reached their highest in early stage spheroidgenesis (8 hours after seeding on ULA plates). At that stage, MCF-7 cells proliferated and formed loose aggregates. After that time, their levels gradually decreased and reached their lowest levels at late stage spheroidgenesis with the formation of tight spheroids. Similarly, the levels of p110γ and class III also increased and reached the highest at 24 hours and then decreased (Figure 6C).

#### Alterations in P13K activity with calpain inhibition

We then asked whether calpains were involved in the regulation of PI3K activity. To address this question, we performed experiments as before with three calpain inhibitors: calpeptin, calpain inhibitor III, and calpain inhibitor IV. In MCF-7 monolayer cells, there was no change in the levels of PI3 Kinase p110α, when treated with either of the three calpain inhibitors (Figure 6D). However, in MCF-7 spheroids, although there was no change in the levels of p-p55 (Tyr199) and p110β (data not shown), there was significant increases in the levels of PI3 Kinase p110α when treated with all three calpain inhibitors (Figure 6D) again implying that the three-dimensional structure of the induced spheroids also influenced P13K activity.

### Observational studies

**Figure 7:**
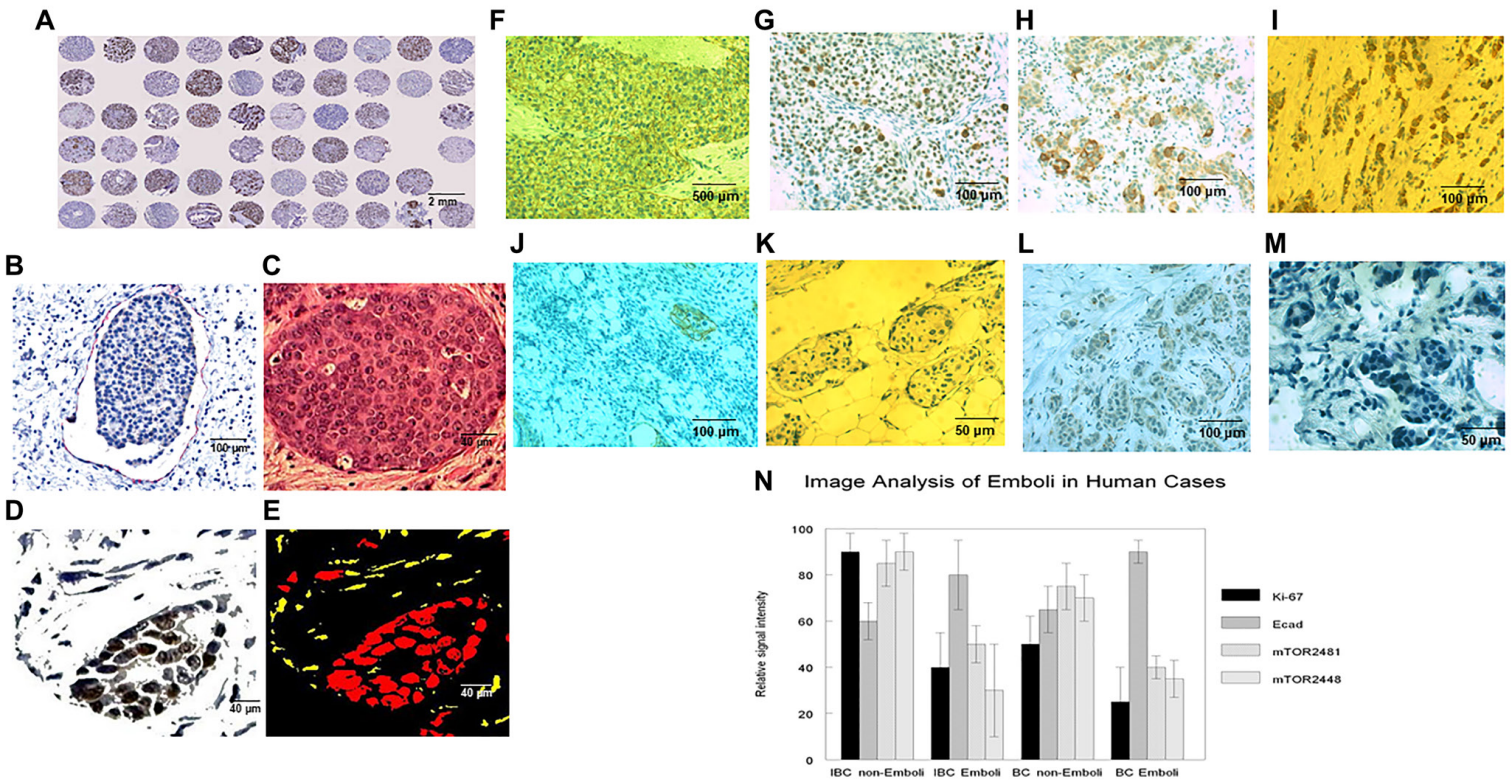
Image and algorithmic analyses of multiple histological and immunocytochemical parameters in emboli *v* non-emboli in IBC and non-IBC cases. TMAs (**A**) were subjected to ERAs and SRAs designed to measure true lymphovascular tumor emboli and distinguish them from tumor clumps showing separation artefact from adjacent stroma (**A**–**C**). Imaging strategy was predicated on SRA’s recognizing podoplanin (D2-40) and CD31 red colorimetric immunoreactivities (B) and the use of ERA’s recognizing epithelial clustering algorithms eg, the Gaussian kernel (**D**), which selectively recognized the clusters within lymphovascular spaces and imaged them (**E**). SRAs showed, compared to their respective non-embolic areas (**F**–**I**), increased E-cadherin (**J**), decreased Ki-67 (**K**), decreased mTOR (Serine2481) (**L**) and decreased mTOR (Serine2448) (**M**) signal intensities within the emboli of both IBC and non-IBC cases (**N**). For each of these parameters, the graph depicts calculated means ± SD (**N**). Differences of significance are depicted.

In observational human studies our image algorithms applied to TMAs of IBC and non-IBC were highly effective in both identifying lymphovascular emboli and comparing them to non-embolic tumoral areas (Figure 7A–7E). There were significant differences in E-cadherin, Ki-67, mTOR (Serine2481) and mTOR (Serine2448) signal intensities with the emboli exhibiting increased E-cadherin (*p* < .05), decreased Ki-67 (*p* < .05), decreased mTOR (Serine2481) (*p* < .05) and decreased mTOR (Serine2448) immunoreactivity (*p* < .01) (Figure 7F–7M). This held true for both IBC as well as non-IBC cases (Figure 7N). These findings corroborated the observations made in both past and present experimental studies respectively on E-cadherin [34] and Ki-67 immunoreactivities of the lymphovascular tumor emboli of Mary-X and its derived spheroids.

## DISCUSSION

The inability to treat overt metastasis, the major cause of both morbidity and mortality in solid cancers, is still the most important challenge faced by present day oncology [1–7]. Metastases, arising from residual disseminated tumor cells (DTCs) or micrometastases, can happen years or even decades after primary tumor treatment because these residual tumor cells enter dormancy and evade therapies [2, 8–12]. Dormant DTCs may reside as small clusters of quiescent cells or alternatively small indolent micrometastases where cellular proliferation is balanced by apoptosis [36]. Although it has been speculated that dormant DTCs can exit dormancy and begin metastatic growth when the microenvironment is altered, we really do not know what governs the release of dormancy. For that matter, we also really do not know what triggers or induces dormancy in the first place. Understanding how DTCs enter and exit dormancy is key to designing potential therapeutic strategies that effectively prevent metastases and recurrence by targeting dormant DTCs [37]. However, the transition process, in which the crosstalk of DTCs with their microenvironment leads to the establishment of or exit from dormancy, is poorly understood [17, 18]. This is largely because it is difficult, if not impossible, to monitor the transition process *in vivo*, and alternately detect and isolate tumor cells in transition from clinical samples. To date, there are no imaging moieties to detect dormant micrometastases in patients and monitor their progression [3]. Moreover, animal models that can recapitulate this transition process are also lacking.

**Figure 8:**
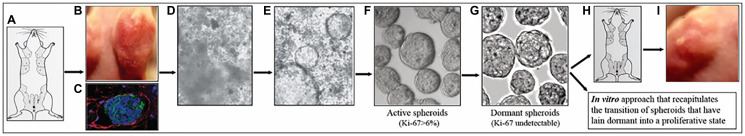
Schematic of the Mary-X xenograft/spheroidgenesis model of tumor dormancy. Schematic depicts the Mary-X xenograft/spheroidgenesis sequence (**A**–**G**) which initiates tumor dormancy *in vivo* within the lymphovascular tumor emboli (C) and *in vitro* during spontaneous spheroidgenesis (D–G). The latter stages of this sequence result in the release of dormancy when the dormant spheroids (G) are reinjected into mice (**H**) with the subsequent emergence of Mary-X (**I**).

Mary-X, a PDX of IBC that was established in our lab [20–23] and its derived spheroids is an ideal model to study the transition of tumor cells from a proliferative to a dormant state and back (Figure 8). Our previous studies had shown that a multi-enzyme cascade of E-cadherin proteolysis was required for the formation of tight structures of both spheroids *in vitro* and lymphovascular emboli *in vivo* [34, 35]. Among those proteases, calpain 2-mediated E-cadherin proteolysis played a key role. Accompanied by E-cadherin proteolysis during their formation, the proliferation index of both the lymphovascular emboli and the Mary-X spheroids specifically decreased over time and a G_0_/G_1_ cell cycle arrest ensued yet the cells did not undergo either apoptosis nor non-apoptotic necrosis and remained viable with the retention of full tumorigenicity. This is the classic definition of dormancy.

Because it would be expected that dormant cancer cells would have reduced metabolism, we investigated the common signaling pathways [38–52] present in cancer that are thought to directly or indirectly regulate growth and metabolism to see whether they played a role in this process. In this study, we investigated the AMP-activated protein kinase (AMPK) pathway, a nutrient-responsive metabolic checkpoint pathway coordinating cell growth with energy status [38–42], the mammalian target of rapamycin (mTOR) pathway, a pathway that is a highly conserved regulator of cell growth found in all eukaryotes [43–48] and the phosphoinositide 3-kinase (PI3K) pathway, a pathway stimulated by diverse oncogenes and growth factor receptors and a pathway generally thought to exhibit increased activity in most cancers [49–52].

Because our previous Mary-X studies had demonstrated the transcriptome equivalence of xenograft-generated spheroids *in vitro* with the lymphovascular emboli *in vivo* with both structures also demonstrating E-cadherin overexpression and specific proteolytic processing producing a number of specific E-cadherin fragments with only calpain 2-generated E-cad/NTF1 present at late spheroidgenesis [34, 35], we also investigated the relationship of calpain-mediated E-cadherin proteolysis to each of these metabolism-regulating pathways in the hopes of gaining insight into the mechanisms of dormancy initiation.

Our experimental studies indicated that the induction of dormancy within both the lymphovascular emboli and end-stage spheroidgenesis of Mary-X was also mediated by calpain 2-mediated E-cadherin proteolysis which triggered decreased P13K signaling, resulting in decreased mTOR activity. This same signaling cascade mediated dormancy in other E-cadherin positive but not negative breast carcinoma cell lines during induced spheroidgenesis.

Interestingly our findings indicated that AMPK, though responsive to calpain inhibition, showed decreased activity in contrast to mTOR which showed increased activity. AMPK activity similarly to mTOR activity was markedly decreased in both spontaneous as well as induced spheriodgenesis. Although increased AMPK activity had been generally thought to inhibit mTOR activity [38–42], this was not the case here because both AMPK and mTOR activities were low at late spheroidgenesis. Although increased AMPK activity and its resultant mTOR pathway inhibition had been regarded to be a response to nutrient deprivation [38–42], in the experiments performed in this study, there was no nutrient deprivation. Therefore the reduced AMPK activity which was observed was not surprising. Since AMPK was not regulating mTOR in the context of calpain-mediated proteolytic processing of E-cadherin, it made sense to investigate other signaling pathways such as P13K.

Calpain inhibitor-regulated activities of both P13K as well as mTOR and AMPK was manifest only in the induced spheroids and not monolayers, implying that the three-dimensional structure of the former was important. This was not the only example of where the three dimensional structure is central to our understanding of what is happening *in vitro* and ultimately *in vivo*. *In vitro* 3D models have been thought to better recapitulate the 3D situation of *in vivo* cancer. *In vitro* 3D models have been thought to more faithfully recapitulate *in vivo* gene expression and other *in vivo* phenomena [53–55]. It was therefore not surprising that *in vitro* 3D models in this case have proved to be a model to study dormancy.

The signaling pathways by which mTOR inhibits cell cycle progression have been incompletely understood. In proliferating cells treated with rapamycin, restoration of mTOR signaling (by using a rapamycin-resistant mutant of mTOR) rescued rapamycin-inhibited G_1_ -phase progression, and restoration of signaling along the mTOR-dependent S6K1 or 4E-BP1/eukaryotic translation initiation factor 4E (eIF4E) pathways provided partial rescue [56]. Thus, in that study, the activities of both the S6K1 and 4E-BP1/eIF4E pathways were required and independently mediated mTOR-dependent G_1_-phase progression [56]. However that study involved proliferating cancer cells in monolayer culture. The mechanisms by which mTOR inhibits cell cycle progression in the context of 3D spheroids or 3D lymphovascular emboli may differ and therefore need to be further explored. In fact E-cadherin proteolysis creating a high density spheroid via E-cad/NTF1 may cause both a cell cycle arrest and mTOR downregulation by separate mechanisms which are completely independent of one another.

It is interesting that even in the setting of *in vitro* 3D models, the hypothesis of epithelial-mesenchymal transition (EMT) still dominates the thinking of the metastatic process even when applied to metastasizing clumps where E-cadherin overexpression is observed [57, 58]. Despite this hypothesis, the evidence suggests that the generation of the lymphovascular embolus does not involve either complete or partial EMT but rather solely E-cadherin overexpression and its proteolysis into fragments that result in increased homotypic adhesion. This results in not only growth arrest, low apoptosis and a decrease in metabolism but suppression of non-apoptotic cell death pathways including ferroptosis [59]. All this initiates tumor dormancy.

## MATERIALS AND METHODS

### Institutional approvals

Collection and use of human breast cancer tissues, completely anonymized, had been approved by The Ohio State University Cancer Institutional Review Board (IRB) under protocol 2006C0042. Additional cases of breast cancer were obtained from and anonymized from the Meharry Medical College Translational Pathology Shared Resource Core, IRB Protocol 23-10-1410.

Mary-X, had been derived from a patient with a biopsy proven diagnosis of IBC in the 1990’s and made into a patient-derived transplantable xenograft (PDX). Studies were conducted under the UCLA’s Human Subject Protection Committee and the Chancellor’s Animal Research Committee (Certification 95-127-11). The xenograft has been phenotypically stable for over 30 years of passage. Most recent animal studies were conducted at Meharry Medical College, OLAW D16-00261 (A3420-01), IACUC protocol 24-02-1443.

### ATCC patent deposits and cell identification

Mary-X and its *in vitro* derived spheroids were deposited in the ATCC cell repository (Manassas, VA, USA) as PTA-2737 and PTA-27376 respectfully and recently verified and re-verified to be both novel and human in origin (STRA4993). Other human E-cadherin positive (MCF-7, T47D) and E-cadherin negative (MDA-MB-468 and MDA-MB-231) cell lines had previously been purchased from ATCC, Manassas, VA, USA.

### Experimental studies

#### Initial xenograft studies

Athymic (nude) mice on BALB/c backgrounds, 4 week old females, purchased from Anticancer, Inc. (San Diego, CA, USA) were derived from their respective breeding colonies.

### *In vitro* studies with Mary-X spheroids and other breast cancer cell lines

Mary-X was placed in culture and gave rise to liberated loose aggregates in suspension culture which then tightened into spheroids over the next 24 hr and remained in suspension culture [20–23]. Spheroids were seeded on 24-well plates in DMEM supplemented with 10% FBS and treated with different inhibitors for a 24-hour period.

Other breast cancer cell lines which included the E-cadherin positive (MCF7, T47D) and negative (MDA-MB-468, MDA-MB-231) cell lines were cultured in Dulbecco’s Modified Eagle’s Medium (DMEM) supplemented with 10% (vol/vol) FBS and 100 U/mL penicillin/streptomycin. These lines grew as monolayers but could be induced to grow as spheroids. 5 × 10^4^ cells were seeded on 24-well Ultra-low Attachment (ULA) plates to induce spheroidgenesis.

### Immunocytochemical experiments on Mary-X and Mary-X spheroids

Mary-X and Mary-X spheroids were subjected to single label immunocytochemical studies measuring both proliferative (Ki-67) (PI) and apoptotic (TUNEL) indices (AI) [25, 26]. Non-apoptotic necrosis was measured by SYTOX Green staining (Thermofisher, Waltham, Massachusetts) and viewed with a Olympus Fluoview-1000 confocal scanning system. 5–10 µm sections of Mary-X were routinely processed for IHC. The Mary-X spheroids were immobilized on glass-bottom dishes coated with Cell-TEK adhesive. The adherent spheroids were then fixed with 4% paraformaldehyde, after permeabilizing with TX-100 and blocking with normal goat serum. Primary antibodies used included rabbit monoclonal primary antibodies to human Ki-67 (RM-9106, 1:100, Epredia) and anti-fluorescein antibodies following incorporation by terminal deoxynucleotidyltransferase (TdT) (Roche Molecular Biochemicals, Mannheim, Germany) [26].

### Cell cycle and flow cytometric studies

Mary-X spheroids were analyzed for cell cycle parameters, generating DNA ploidy fluorescence histograms. l0^6^ cells from the spheroids were harvested in trypsin-EDTA, suspended in I ml of hypotonic staining buffer (0.1 mg/mI propidium iodide (Calbiochem), 0.3% Triton X-lOO, 20 mg/ml RNase A (Sigma Chemical Co.) and I mg/ml sodium citrate and analyzed on a FACScan (Becton Dickinson, Mountain View, CA, USA) [27].

### Inhibitors and antibodies

The inhibitors (pathways) used included rapamycin (mTOR), U0126 (MAPK), LY294002 (P13K) and calpeptin, inhibitors III and IV (calpain), all purchased from Fisher Scientific (Waltham, MA, USA). We used the following antibodies for Western blot studies: PI3K Ab Sampler Kit (1:1000 dilution, Rabbit, #9655, Cell Signaling Technology (CST)), AMPK and ACC Antibody Sampler Kit (1:1000 dilution, Rabbit, #9957, (CST)), mTOR Substrates Antibody Sampler Kit (1:1000 dilution, Rabbit, #9862, (CST)), E-Cadherin (24E10) (1:1000 dilution, Rabbit mAb #3195, (CST)) and Calpain 2 Large Subunit (M-type) (1:1000 dilution, #3195, (CST)).

For inhibitor treatment, the inhibitor stock solutions were made in DMSO with 1000× of working concentrations. The inhibitors were added 24 hours after seeding and both the spontaneous spheroids of Mary-X and the induced spheroids of the other cell lines were treated. All the spheroids were then harvested for western blot analysis.

### Western blot analysis

The collected cells or spheroids were washed in cold PBS and then suspended in Laemmli Sample Buffer (#1610737. Bio-Rad, Hercules, CA, USA) with β-mercaptoethanol and boiled for 10 min. Whole-cell lysates were separated by sodium dodecyl sulfate-polyacrylamide gel electrophoresis on precast 4–20% Mini-Protean TGX gels (Bio-Rad), transferred to PVDF membranes, and probed with the indicated antibodies.

### ImageJ analysis

Quantification of protein band intensities were performed with ImageJ Software (NIH) [28].

### Statistical analysis

For PI and AI, means ± SD values were determined. All experiments were performed by counting 100 spheroids. For the Western blot studies, all experiments were performed in quadruplicate and band intensities compared. All stated or calculated differences implied differences of statistical significance, assessed by the two tailed students *t*-test as well as ANOVA.

### Observational studies

#### Cases of IBC and non-IBC

100 cases of IBC had been randomly selected from a database and the Ohio State University’s Information Warehouse and anonymized. 25 cases of non-IBC showing prominent lymphovascular invasion were obtained from the Meharry Medical College and its Translational Pathology Shared Resource Core.

### TMA construction

Multiple 2-millimeter tissue cores of tumor from each paraffin-embedded donor block (average of 10 cores/block) were arrayed into recipient TMA blocks. Our specific TMA algorithms carried out virtual alignment, image processing, and the application of the epithelial recognition algorithms (ERAs) and specific recognition algorithms (SRAs) which recognized lymphovascular tumor emboli within lymphovascular channels, the latter based upon immunocytochemical studies [25]. Additional SRAs based on nuclear, membrane and dual cytoplasmic and membrane compartmental immunoreactivities quantitated their respective signal intensities [29–32] utilizing ImageJ Software [28, 33]. Image acquisition was by the iSCAN System (BioImagene, Inc, Cupertino, CA, USA).

### Histological and immunocytochemistry studies

Primary antibodies used included D2-40 anti-podoplanin (clone D2-40, Dako, catalog number M3619), anti-CD31 (rabbit polyclonal, Spring Bioscience, catalog number E11114), rabbit monoclonal primary antibodies to human Ki-67 (RM-9106, 1:100, Epredia) and E-Cadherin (3195. 1:400), phospho-mTOR(Ser2481), Ab #2974 and phospho-mTOR(Ser2448), Ab #2971 (CST).

### Image analysis comparing E-cadherin, Ki-67, mTOR (Serine 2481, 2448) relative signal intensities in lymphovascular tumor emboli *v* non-embolic areas

ERAs applied to each TMA core were successful in recognizing tumor emboli based on the Gaussian kernel and specific circumferential lymphovascular immunoreactivities demonstrated previously [25]. Other SRAs quantitated nuclear Ki-67, membrane E-cadherin and mTOR (Serine2481, 2448) dual compartment (cytoplasmic and membrane) immunoreactivities [29–32].

### Statistical analysis

For E-cadherin, Ki-67, mTOR (Ser248, Ser2448), relative signal intensity in the embolic *v* non-embolic areas were expressed as means ± SD values. 100 emboli from each IBC and 10 emboli from each non-IBC case were analyzed. All experiments were performed in quadruplicate. All stated or calculated differences implied differences of statistical significance, assessed by the two tailed students *t*-test as well as ANOVA.

## Abbreviations

IBC: inflammatory breast cancer
EMT: epithelial-mesenchymal transition
ERAs: epithelial recognition algorithms
SRAs: specific recognition algorithms
TMA: tissue microarray
ANOVA: analysis of variance
PDX: patient-derived xenograft
E-cad/NTF-1: E-cadherin/N-terminal fragment-1
PI: proliferative index
AI: apoptotic index
mTOR: mammalian target of rapamycin
AMPK: 5’ AMP-activated protein kinase
PI3K: phosphoinositide 3-kinase
ACC: acetyl-CoA carboxylase
DTC’s: disseminated tumor cells
